# High Definition 3D Map Creation Using GNSS/IMU/LiDAR Sensor Integration to Support Autonomous Vehicle Navigation

**DOI:** 10.3390/s20030899

**Published:** 2020-02-07

**Authors:** Veli Ilci, Charles Toth

**Affiliations:** 1 Department of Geomatics Engineering, Ondokuz Mayis University, 55139 Atakum, Turkey; 2 Department of Civil, Environmental and Geodetic Engineering, Ohio State University, Columbus, OH 43210, USA; toth.2@osu.edu

**Keywords:** autonomous vehicle, mobile mapping, sensor fusion, point cloud, LiDAR

## Abstract

Recent developments in sensor technologies such as Global Navigation Satellite Systems (GNSS), Inertial Measurement Unit (IMU), Light Detection and Ranging (LiDAR), radar, and camera have led to emerging state-of-the-art autonomous systems, such as driverless vehicles or UAS (Unmanned Airborne Systems) swarms. These technologies necessitate the use of accurate object space information about the physical environment around the platform. This information can be generally provided by the suitable selection of the sensors, including sensor types and capabilities, the number of sensors, and their spatial arrangement. Since all these sensor technologies have different error sources and characteristics, rigorous sensor modeling is needed to eliminate/mitigate errors to obtain an accurate, reliable, and robust integrated solution. Mobile mapping systems are very similar to autonomous vehicles in terms of being able to reconstruct the environment around the platforms. However, they differ a lot in operations and objectives. Mobile mapping vehicles use professional grade sensors, such as geodetic grade GNSS, tactical grade IMU, mobile LiDAR, and metric cameras, and the solution is created in post-processing. In contrast, autonomous vehicles use simple/inexpensive sensors, require real-time operations, and are primarily interested in identifying and tracking moving objects. In this study, the main objective was to assess the performance potential of autonomous vehicle sensor systems to obtain high-definition maps based on only using Velodyne sensor data for creating accurate point clouds. In other words, no other sensor data were considered in this investigation. The results have confirmed that cm-level accuracy can be achieved.

## 1. Introduction

An autonomous vehicle (AV) is a self-driving car that has a powerful real-time perception and decision-making system [1]. The Society of Automotive Engineers has defined the levels of AVs from level 0 (no automation) to level 5 (fully automated) [2]. Although the automotive industry leaders, information and communication technology companies, and researchers aim for fully automated vehicles to participate in the emerging market of autonomous vehicles [3], currently, most commercially available vehicles use advanced driver assistance systems (ADAS) support level 2 and only a few recently introduced vehicles have Level 3 performance. Vehicle-to-roadside-infrastructure (V2I) and vehicle-to-vehicle (V2V) communications help to improve traffic safety and autonomous driving functionality [4]. It is expected that fully automated cars will be commercialized, and will appear on the roads in the coming years [5], will prevent driver-related accidents [6], and will decrease transportation problems such as regulating traffic flow [4]. Perception, localization and mapping, path planning, decision making, and vehicle control are the main components of AV technology [7]. For common use, AVs must be robust, reliable, and safe enough for real-world conditions. AVs must have advanced sensing capabilities to continuously observe and perceive their surroundings as well as to accurately calculate their location on a global scale and relative to the other static or dynamic obstacles [7]. Also, AVs have to follow all of the safe driving rules.

Two main techniques to navigate available for AVs technology are Simultaneous Localization and Mapping (SLAM) and High Definition (HD) maps. In the SLAM method, perception and localization are completed in real time. SLAM-based techniques continuously monitor the environment and easily adapt to new cases. These techniques require more computationally intensive algorithms and may be subject to more uncertainty depending on the sensors used and on the surroundings [7]. Due to the limitations of the sensors, such as perception range and recognition performance, AVs cannot detect distant objects or objects blocked by obstacles in real time [6]. Using HD maps overcomes these limitations and offers a detailed representation of the surroundings of the AV, and thus, the perception task of AV systems is significantly assisted; it is much easier to find and identify objects if they are known. HD maps are useful if the physical environment does not often change; however, if significant changes occur, this technique may lead to unexpected problems for autonomous driving. Thus, HD maps have to be kept up to date to provide a sustained performance level for the real-time perception of AVs to precisely localize themselves. Also, the large size of the storage data, computational load [8], and transmission latency are currently the main drawbacks of the HD map technology as well as the worldwide availability of HD maps. To reduce the storage and computational load, the necessary part of the HD map may be loaded to the AV. In summary, AVs use the HD maps and combine the map with the self-localization solution to simplify the perception and scene-understanding problem [9].

In the last two decades, unprecedented improvements in sensor technologies and data processing methods have led to the emergence and rapid development of state-of-the-art technologies, such as autonomous vehicles (AV) and mobile mapping systems. In particular, car manufacturers and IT (Internet Technology) giants have been devoting significant research and development efforts to building fully automated vehicles and to making them commercially available within the next decade [10]. Accurate position and attitude information of the vehicle as well as the ability to detection static and dynamic obstacles around it are among the crucial requirements of autonomous vehicle technology that is a particularly challenging in urban areas [11,12]. Furthermore, autonomous vehicle technologies require all this information in real time, which poses an implementation problem due to current limitations of the computer capacity, processing complexity, transfer capability to the cloud [13], etc. Mobile mapping systems produce 3D high-definition information of the surrounding of the platform by integrating navigation/georeferencing and high-resolution imaging sensor data [14,15]. Autonomous vehicle technology requires real-time sensor data processing, object extraction and tracking, and then scene interpretation and finally drive control. Using map-matching algorithms to obtain more consistent, accurate, and seamless geospatial products can provide essential help for localization [16].

In mobile mapping systems, the position and the attitude information of the moving platform must be known as accurately as possible. Global Navigation Satellite Systems (GNSS) are the primary navigation/georeferencing technology offering high positioning accuracy in the open areas [17]. Unfortunately, in partially or entirely GNSS-compromised environments, such as urban areas or tunnels, the provided accuracy of the GNSS system degrades dramatically [18,19]. Inertial Measurement Unit (IMU) can provide relative attitude and position information of the mobile platform at high data rates. IMU-based accuracy, however, degrades quickly over time [20] due to the inherent drift depending on the quality of the IMU [21] unless positioning fixes are provided from another source. Integrating GNSS and IMU technologies are used to compensate for standalone deficiencies and to provide better accuracy, reliability, and continuity of the navigation solution [22]. The imaging sensors of mobile mapping systems are time synchronized to GPS time, and thus, the platform georeferencing solution can be easily transferred to imaging data streams, so data can be fused and projected to a global coordinate system [23].

In recent years, emerging light-weight and small size sensors have provided an opportunity to use multiple sensors on mobile platforms, typically including GNSS, IMU, LiDAR, cameras, and ultrasonic sensors [24]. While using multiple sensors provides rich sensing capabilities, expressed in redundant and/or complementary observations, different data acquisition methods, different error properties, uncertainty in data sources, etc. pose challenges for data fusion [25]. Accurate individual and inter-sensor calibration of the sensors have to be achieved; the position and attitude of each sensor have to be defined in a common reference frame [26]. On classical mobile mapping platforms, the IMU frame is used as a local reference system, and thus, the georeferencing solution is calculated in this frame. Using conventional surveying techniques, the lever arms are determined at good accuracy with respect to the IMU frame. Boresight misalignment, however, requires indirect estimation as attitude differences cannot be directly measured at the required accuracy level [27]. Using inexpensive sensors makes these calibration processes quite challenging and is an active area of research along with the data processing [28].

Most of the AV sensing is based on using point clouds, typically obtained by laser sensors or produced by stereo/multiray image intersections. Consequently, point cloud matching is one of the fundamental elements of low-level perception. The iterative closest point (ICP) method is a well-known scan-matching and registration algorithm [29] that was proposed for point-to-point registration [30] and point-to-surface registration [31] in the 1990s to minimize the differences between two point clouds and to match them as closely as possible. This algorithm is robust and straightforward [32]; however, it has some problems in real-time applications such as SLAM due to heavy computation burden [33,34] and huge execution time [35]. Also, sparse point clouds and high-speed moving platforms introducing motion distortion can affect the performance of this algorithm negatively [36]. Many improvements have been proposed [37,38] to mitigate the limitation and to improve the computation efficiency and accuracy of the ICP algorithm [39].

Aside from ICP, Normal Distributions Transform (NDT) was introduced by Biber and Strasser in 2003 for scan matching and registration of laser-scan data. In NDT, the reference point cloud is transformed into fixed 2D cells and is converted to a set of Gaussian probability distribution, and then, scan data is matched to the set of normal distributions [40]. In other words, NDT is a grid-based representation that matches LiDAR data with the set of normal distributions rather than point cloud. Drawbacks of the NDT algorithm is the sensitivity to the initial guess. The matching time of the NDT is better than ICP because NDT does not require point-to-point registration [34]. However, the determination of the grid size is a critical step in this algorithm, which is an issue for inhomogeneous point clouds [41] that dominate the estimation stability and determines the performance of the algorithm [35]. This algorithm has been used for many applications, such as path planning, change detection, and loop detection [32]. Furthermore, this method cannot adequately model the positioning uncertainty caused by moving objects [42].

The LiDAR Odometry and Mapping method (LOAM) has been proposed by Zhang and Singh in 2015 to estimate accurate motion and mapping in real-time. The LOAM is a combination of two algorithms. The LiDAR odometry carries out course processing to determine the velocity at a higher frequency, and LiDAR mapping performs fine processing to create maps at a lower frequency [43]. Increasing drift error over time that is not corrected is the main drawback of this algorithm because of no loop closure detection [35]. Particularly, the performance of this algorithm severely degrades if the number of moving objects increases, such as in urban areas [42].

## 2. Motivation

A broad spectrum of various grade imaging/mapping and navigation sensors are used in mobile mapping systems [44,45,46] and autonomous vehicles technologies [47,48,49]. In particular, the second application is experiencing an explosive growth recently, and the problem is generally posed as how to select the minimum number of inexpensive sensors to achieve the required level of positioning accuracy and robustness of vehicle navigation in real time, as an important aspect is the affordability, e.g., AVs need to be competitive in pricing to regular stock vehicles. In contrast, the objective of mobile mapping systems is to acquire a complete representation of the object space at very high accuracy in post-processing. Therefore, these systems use high-grade sensors, which are not prohibitive for these applications.

Both systems, AV and mobile mapping, are based on sensor integration, and to achieve optimal performance with respect to the sensor potential, careful system calibration must be performed. In the general approach, all the sensors should be individually calibrated in a laboratory environment and/or in situ; if feasible, the second option is preferred, as it could provide the most realistic calibration environment. To form the basis for any sensor integration, the sensor data must be time synchronized and the sensors’ spatial relationship must be known. The time synchronization is typically an engineering problem, and a variety of solutions exists [50,51,52]. The spatial relationship between any two sensors, in particular, the rotation between two sensor frames, is of critical importance as it cannot be directly measured, compared to distances between sensors which can be easily surveyed. The overall performance analysis of highly integrated AV systems is even more complex as, during normal vehicles dynamics, such parameters may change, noise level may vary, scene complexity impacts real-time processing, etc.

To perform a performance assessment of AV systems, either a reference solution is needed or adequate ground control must be available in an area where the typical platform dynamics can be tested. None of these are simple; having a reference trajectory would require an independent sensor system with an accuracy level of one order higher. Furthermore, the main question for an existing system is how to improve the performance when only the overall performance is known as well as the manufacturer’s sensor specification parameters. There is no analytical error budget, so it is not obvious to decide which sensor should be upgraded to a better grade. This investigation considers a specific case of an AV system, a LiDAR sensor with navigation sensors, and the objective is to determine the performance potential of the LiDAR sensor in normal operations. Furthermore, a high-end georeferencing system is used in order to obtain the LiDAR sensor performance. Of course, the object space may have a strong influence on the error budget, as reflections can vary over a large range. Since AV technology is primarily deployed in urban environments, most of the objects around the vehicle are hard surfaces with modest fluctuation in reflectivity; this aspect is not the subject of the investigation here.

In summary, the objective of this study is to assess the feasibility of creating a high-definition 3D map using only auto industry-grade mobile LiDAR sensors. In other words, can LiDAR sensors deployed on AV create accurate mapping of the environment, the corridor the vehicle travels? AV experts agree that having an HD map (high-definition map in automotive industry terms) is essential to improving localization and navigation of AVs. Creating these HD maps by AVs would represent an inexpensive yet effective crowdsourcing solution for AV industry. To support the experimental evaluation, high-end navigation and multiple Velodyne sensors were installed on a test vehicle. The position and attitude information of the vehicle was determined with the direct georeferencing method integrating GNSS and navigation-grade IMU sensor data. The reason why the highest grade IMU was used in this study is that we wanted to obtain the most accurate platform georeferencing to provide a high-quality reference for the sensor performance evaluation. Seven LiDAR sensors with cameras collected 3D geospatial data around the platform. The Pulse Per Second (1PPS) signal and National Marine Electronics Association (NMEA) messages from the Novatel GNSS receiver were used to provide time synchronization for the LiDAR sensors. The focus of our effort was only on the LiDAR data streams, namely how they can be merged into a combined point cloud and be transformed into a global coordinate system. For quality assessment, benchmark ground control data was collected from horizontal and vertical surfaces using classical surveying methods. The performance of the georeferencing solution essential for obtaining performance evaluation of the LiDAR sensors and the quality of the high definition 3D map are investigated and analyzed.

## 3. Data Acquisition System and Test Area

**Figure 1:**
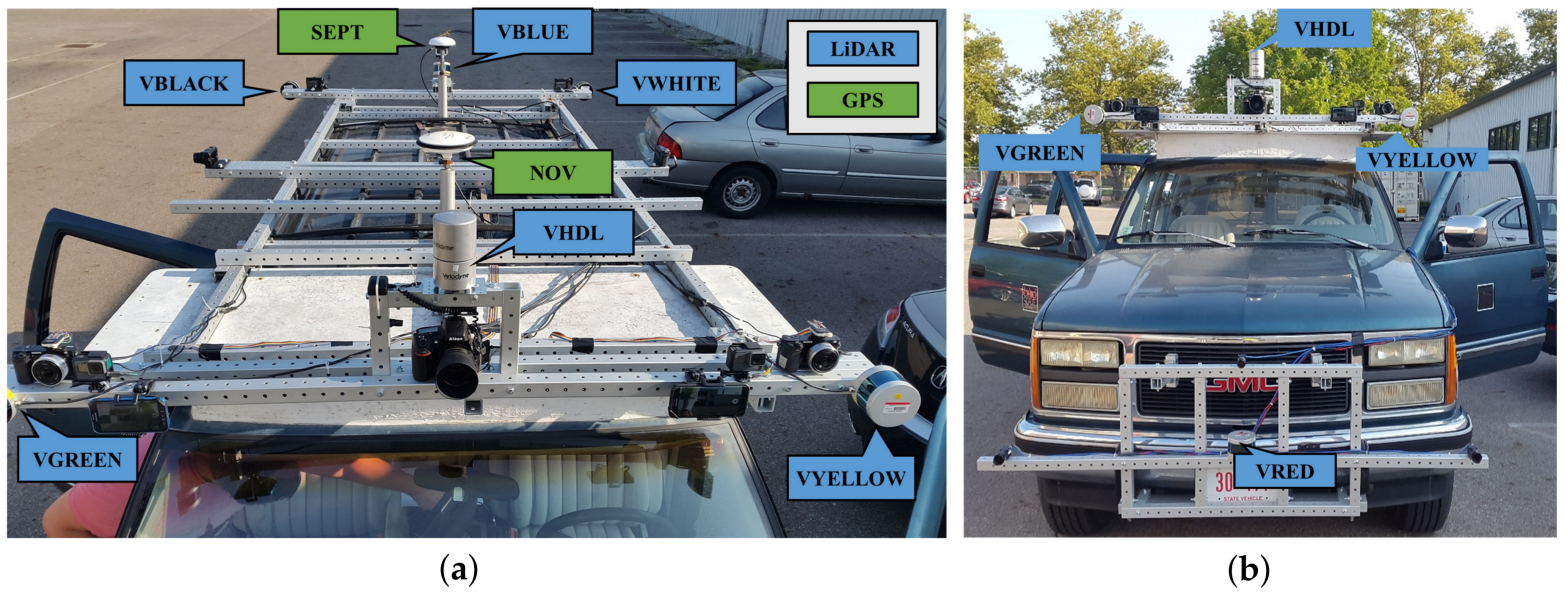
The carrier platforms with the sensor arrangement: (**a**) top view (**b**) front view.

In this study, a GMC Suburban (GPSVan) [53] was used as a moving platform, and two light frames were mounted at the front and at the top of the vehicle. The sensor installation included one Septentrio PolaNt-x MC GNSS antenna, one Novatel 600 antenna GPS antenna, one Velodyne HDL-32E, and five Velodyne VLP-16 LiDAR sensors on the top platform and one VLP-16 LiDAR on the front platform. Note that the cameras are not listed here as they are not part of this study. The Septentrio Rx5 GNSS receiver, Novatel DL-4 GPS receiver, two navigation-grade H764G IMUs, and one tactical-grade IMU were located inside the vehicle. Figure 1 shows the sensor installation on the platform, and the model and location of the sensors with brief technical specifications about the sensors are given in Table 1.

**Figure 2:**
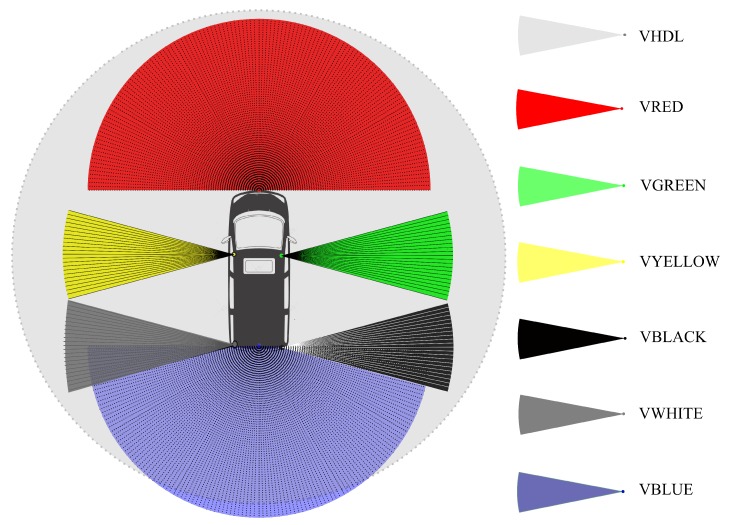
Field of views of the LiDAR sensors around the GPSVan.

The lever arm offsets between GNSS antennas, IMU body, and LiDAR sensors were accurately surveyed in advance and used in georeferencing and boresighting processes later. The position and orientation of the LiDAR sensors were designed to cover the largest field of view (FOV) of the surrounding of the platform. The intended FOV information of the LiDAR sensors is shown in Figure 2.

**Figure 3:**
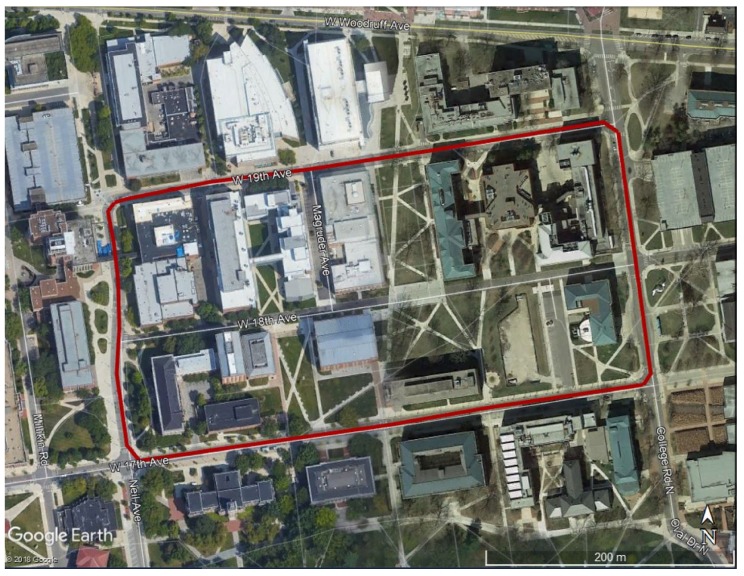
Test area (red line shows the GPSVan trajectory).

Several measurements were carried out at the Ohio State University’s main campus area, Columbus, U.S.; here, the 13 October 2017 session is considered. The area with the test trajectory, shown in Figure 3, is mixed-urban, including densely packed and tall buildings, and roads shared by cars, bicycles, and pedestrians. During the data acquisition, the speed of the platform varied at times due to the presence of 18 crosswalks, three intersections, and one curve along the trajectory. The overall length of one loop is about 1250 meters, and the mean velocity of the platform was 22 km/h. The test measurements were performed six times in the test area to obtain multiple samples and to thus help produce realistic statistics.

## 4. Platform Georeferencing and Inter-Sensor Calibration

### 4.1. Methodology

In mobile mapping systems, data from all of the sensors must be transformed from the sensor coordinate system to a global or common coordinate system [54]. Three-dimensional homogenous point coordinates provided from raw LiDAR sensor measurements are defined in the sensor coordinate system as $x_{s}={\left[\lambda_{X}\lambda_{Y}\lambda_{Z}\lambda \right]}^{T}\epsilon R^{4},\lambda \neq 0$ and transformed from the sensor coordinate system to a global coordinate system as follow:

(1) $$x_{g}=T_{p}^{g}T_{s}^{p}x_{s}$$

where $T_{s}^{p}$ is the time-independent sensor-to-platform transformation matrix which is called boresighting and is defined as follows:

(2) $$T_{s\{4x4\}}^{p}=\left(\begin{array}{cc} R_{s\{3x3\}}^{p} & l_{s\{3x1\}}^{p}\\ 0_{\left\{1x3\right\}} & 1 \end{array}\right)$$

where $R_{s}^{p}$ is the sensor-to-platform rotation matrix, also known as boresight misalignment, and $l_{s}^{p}$ is sensor-to-platform lever-arm offset vector. $T_{p}^{g}$ is the time-dependent platform-to-global transformation matrix which is obtained from GNSS/IMU integration, called georeferencing solution and defined as follows:

(3) $$T_{p\{4x4\}}^{g}=\left(\begin{array}{cc} R_{p\{3x3\}}^{g} & p_{\left\{1x3\right\}}\\ 0_{\left\{1x3\right\}} & 1 \end{array}\right)$$

where $R_{p}^{g}$ is the rotation matrix from the platform coordinate system to the global coordinate system and where *p* is the position vector of the platform.

### 4.2. Georeferencing Solution

Accurate georeferencing of a mobile platform is a crucial prerequisite for any geospatial data processing. In this study, the collected GNSS data, acquired by the Septentrio GNSS receiver and a geodetic grade H764G IMU data, were processed to obtain an accurate georeferencing of the vehicle. Note that the Novatel GPS was only used for the time synchronization of the imaging sensors and that the second H764G IMU and the lower-grade IMUs were used for comparative studies. The nearby Columbus (COLB) station of the National Oceanic and Atmospheric Administration (NOAA) Continuously Operating Reference Station (CORS) network was used as a reference station, and the processing interval and elevation angle for the Septentrio data was set to 10 Hz and 10 degrees, respectively. The differential GNSS process using the NovAtel Inertial Explorer was carried out to obtain both forward and backward solutions, and the solutions were combined to enhance positioning performance. Next, a loosely coupled integration model was used to integrate the GNSS solution and the IMU data to obtain a continuous attitude and position of the platform trajectory. Finally, both forward and backward solutions were combined and smoothed to generate the highest quality trajectory possible [55].

**Figure 4:**
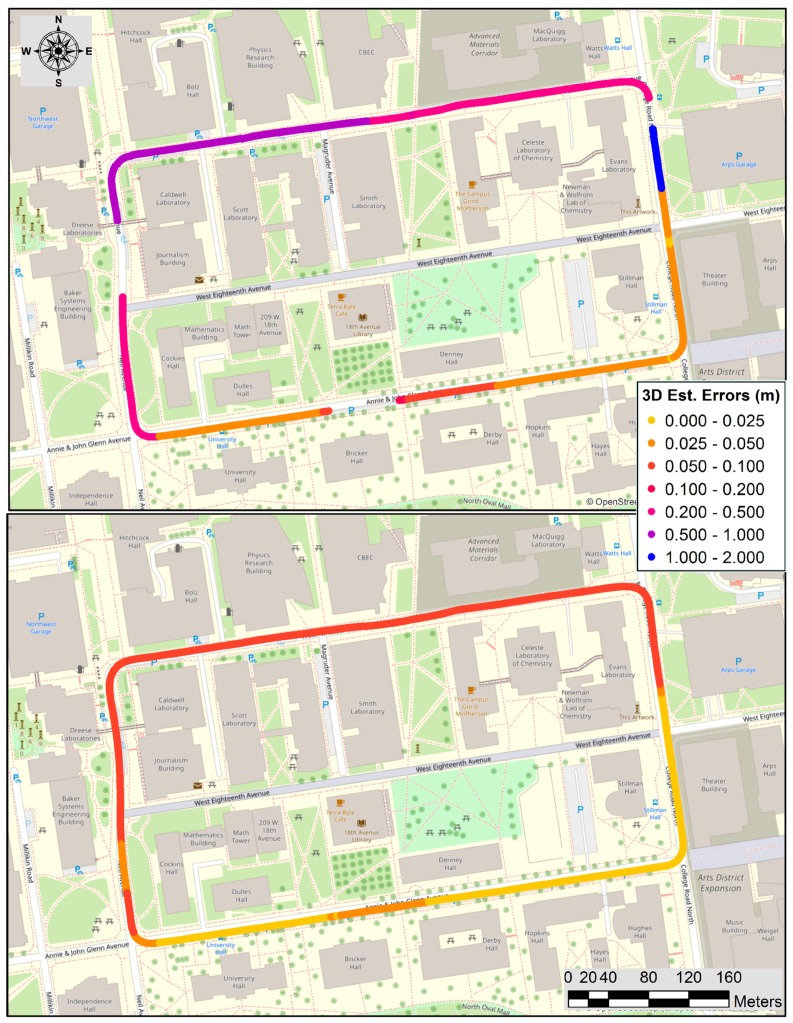
Georeferencing solutions through the trajectory for the 3rd loop; left: GNSS only; right: GNSS/IMU integration (colors in the legend indicate 3D estimated errors in meter).

Figure 4 shows the georeferencing solution of the GNSS only and GNSS/IMU loosely coupled integration solutions for the 3rd loop; the other five loops show similar results. In the GNSS only solution, the northwestern, northeastern, and southern parts of the trajectory have gaps, where the quality of this solution is mostly at the meter level. The urban-canyon effect due to the tall buildings causes signal blockage and the multipath that could significantly decrease the solution quality. Clearly, the GNSS/IMU solution not only bridges the gaps but also improves the accuracy of the positioning and attitude data.

**Figure 5:**
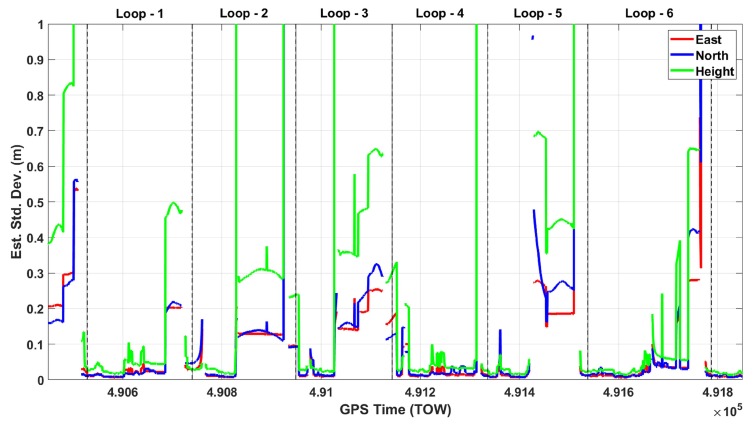
Estimated east, north, and height standard deviations of the GNSS only solution.

**Figure 6:**
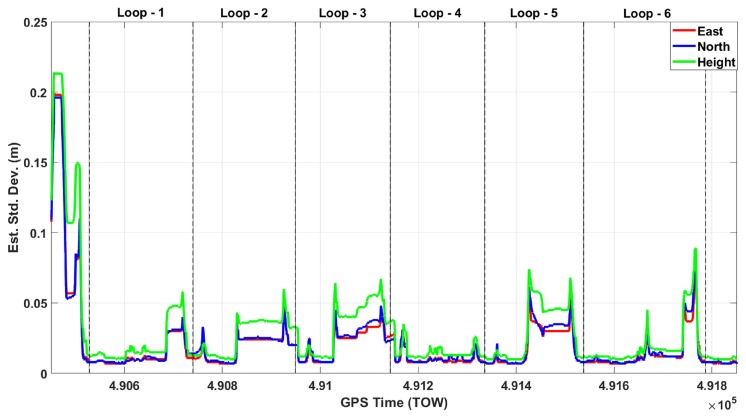
Estimated east, north, and height standard deviations of the GNSS/IMU integration.

**Figure 7:**
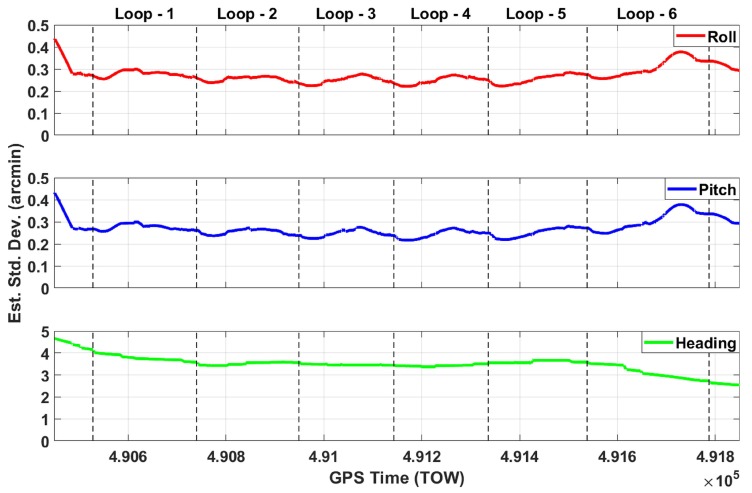
Estimated standard deviations of the roll, pitch, and heading angles of the GNSS/IMU integration.

Figure 5 shows the estimated standard deviations of the GNSS only solution through six loops for east, north, and height. The gaps are clearly seen in this figure in all of the six loops, and estimated standard deviations reach up to the meter level many times in all components. Figure 6 shows the estimated east, north, and height accuracies of the GNSS/IMU solution. This figure clearly demonstrates that the fusion of GNSS/IMU provides a cm-level solution in all components without any gaps through the six loops. The estimated standard deviations of the roll, pitch, and heading components of the GNSS/IMU solution, shown in Figure 7, are at the arcmin level.

Table 2 summarizes the overall georeferencing performance for both the GNSS only and GNSS/IMU solutions. The GNSS only solution provides 8-, 9-, and 20-cm accuracies on average in the east, north, and height components, respectively, but maximum errors reach up to 7.3, 4.0, and 17.0 meters, respectively. On the other hand, the GNSS/IMU integrated solution provides 2-cm accuracy on average in east, north, and height components, and maximum errors remain cm-level in the three components. The average estimated standard deviations of the roll, pitch, and heading components are 0.27, 0.26, and 3.48 arcmin in the roll, pitch, and heading components, respectively. Also, the maximum values reach up to 0.38, 0.38, and 4.13 arcmin levels, respectively. Clearly, the use of navigation-grade IMU has great importance in obtaining these high-level accuracies, which is required to adequately evaluate the point cloud performance. Obviously, this quality IMU is not affordable not only for AV applications but also for mobile mapping systems, where a tactical-grade IMU is typically used. Using lower/consumer-grade IMUs, such as Microelectromechanical Systems (MEMS) is a topic on its own, and there are many publications available in this area.

### 4.3. Boresighting Estimation

**Figure 8:**
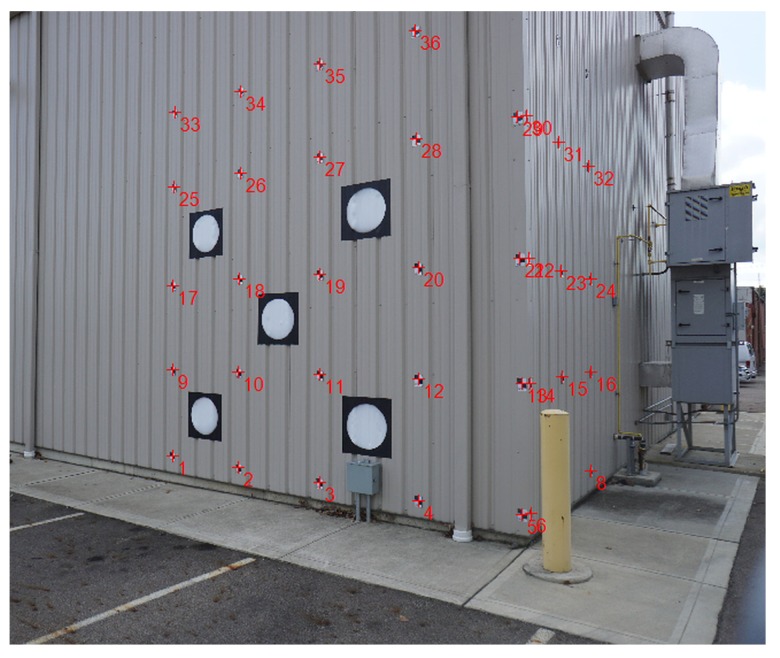
LiDAR targets.

A test range was created at the main facility of the Ohio State University (OSU) Center for Automotive Research (CAR) to determine the boresight misalignment parameters of the sensors installed in the GPSVan. Five LiDAR targets which were large circles in a square with different reflectivities, shown in Figure 8, were attached to the walls, and the target locations were measured using a total station and then tied to the global system using GNSS measurements. LiDAR data sets were acquired from various directions at different ranges. The estimation process used the lever arms as fixed parameters as they were accurately measured by conventional surveying methods, and only the boresight misalignment, $R_{s}^{p}$, in Equation (2), was estimated. The detailed information about the adopted LiDAR boresighting and sensor calibration processes can be found in Reference [54]. Using the calculated boresight misalignment parameters and lever arm values, all the sensor data from the sensor coordinate system was transformed to the platform coordinate system.

## 5. Point Cloud Generation and Performance Analysis

**Figure 9:**
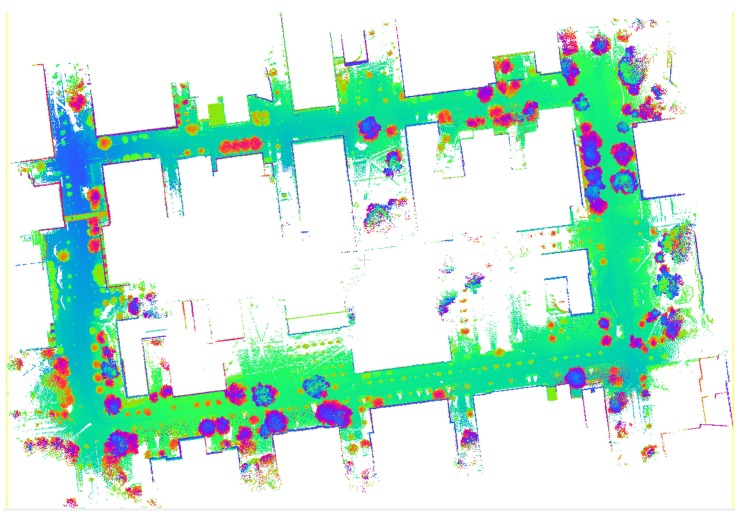
Three-dimensional point cloud generated by all LiDAR sensors.

The MATLAB programming platform was used for the integration of georeferencing and boresighting data to produce the 3D point cloud in 0.1-second intervals. Since the 360${}^{\circ }$ FOVs of the Velodynes had significant overlap and the reflective chassis of the vehicle caused the creation of false points, a filtering window to restrict the FOV was applied during the point generation to obtain clean point clouds. LiDAR point clouds were created from each raw sensor data, were subsequently merged into a single point cloud, and were visualized using the CloudCompare open-source processing software [56]; see Figure 9.

**Figure 10:**
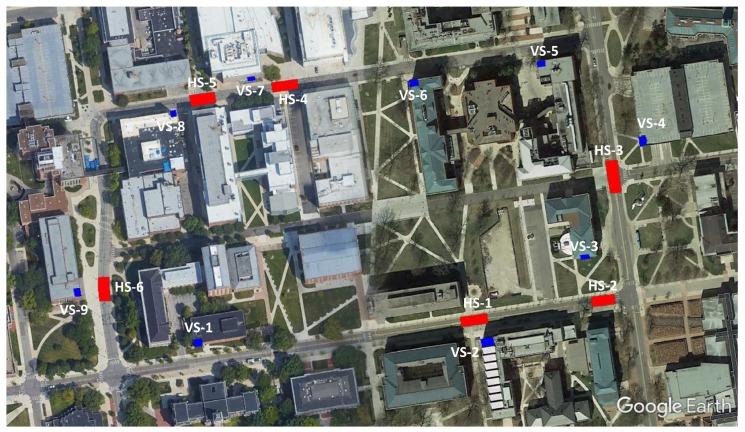
Checkpoints and reference surface locations at the OSU main campus (HS: horizontal surfaces, VS: vertical surfaces).

**Figure 11:**
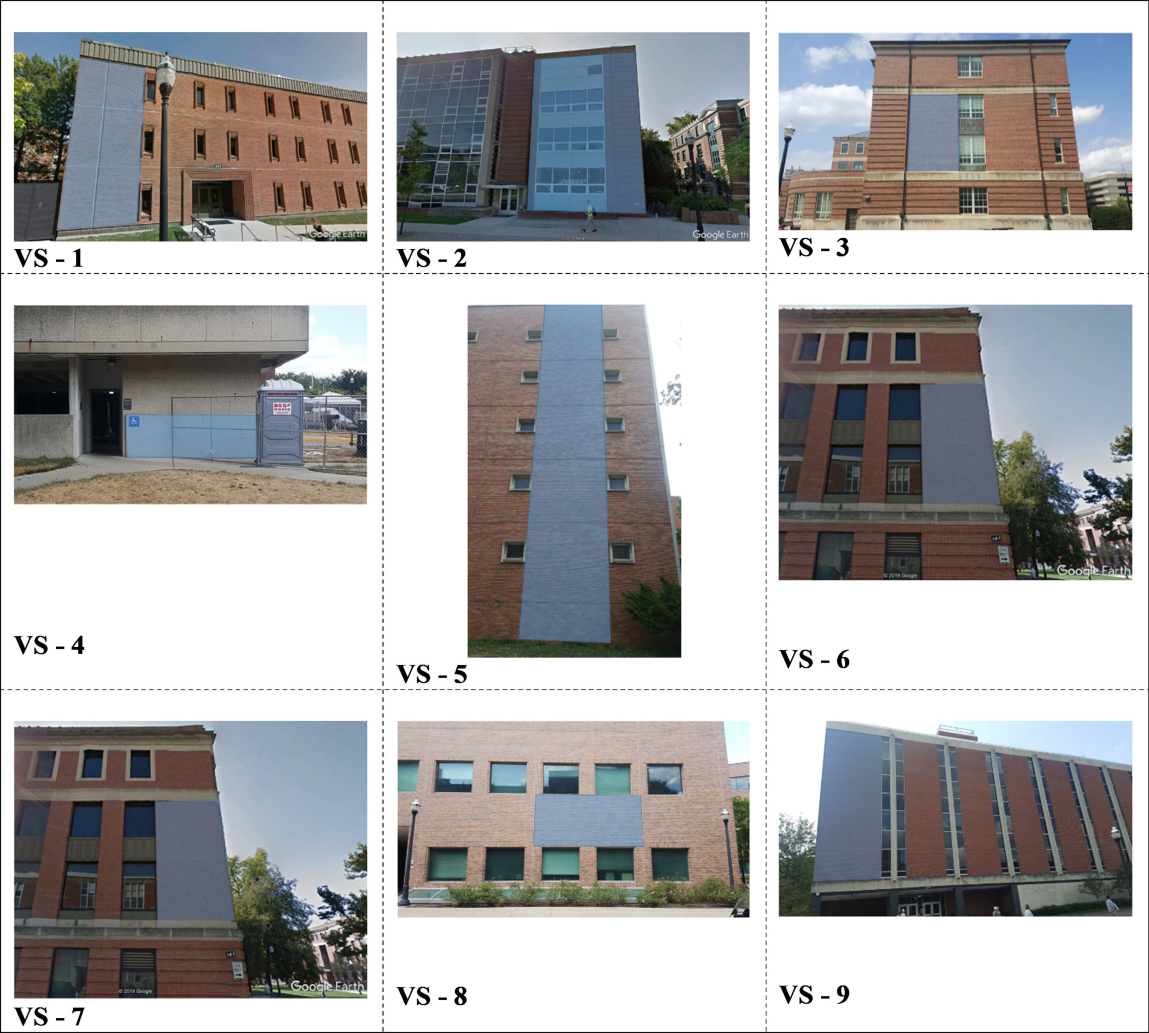
Vertical reference surfaces.

**Figure 12:**
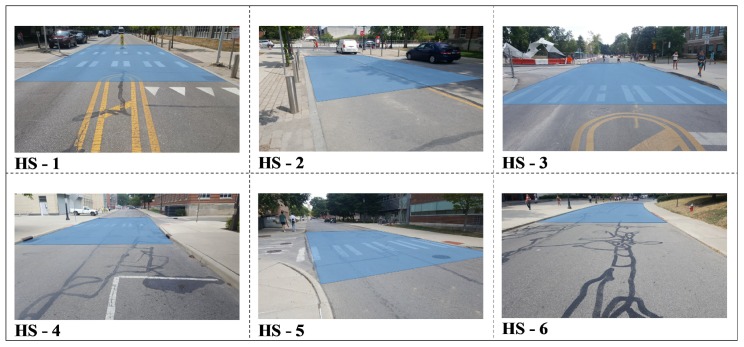
Horizontal reference surfaces.

To perform the quality assessment, including a check on data integrity and absolute accuracy, checkpoints and reference surfaces were established in the area; using GNSS and total station measurements, nine vertical surfaces from building walls and six horizontal reference road surfaces were surveyed. Figure 10 shows the location of the checkpoints and reference surfaces on the OSU main campus. Figure 11 and Figure 12 visualize the reference vertical and horizontal surfaces, respectively. In order to produce the reference data of the surfaces, 11 benchmark points along the loop were established by collecting at least one-hour long static GNSS observations using a Topcon HiperLite dual-frequency GNSS receiver. Three-dimensional coordinates of benchmark points were obtained using the Online Positioning User Service (OPUS) [57]; the overall 2D/3D accuracies of points were 1 cm and 2 cm, respectively. With respect to the 11 reference points, the coordinates of the reference surface points were obtained by terrestrial measurements using a Leica TS06 plus total station.

The CloudCompare software was the primary tool to compare the LiDAR 3D point cloud to the reference point clouds at both the horizontal and vertical surfaces. For the horizontal surfaces, to model the road shape, the quadric polynomial based comparison was adopted, while for vertical surfaces and building walls, a simple plane-fitting based comparison was used. The residuals of reference surfaces are listed in Table 3. The mean differences at vertical surfaces change between 0 and 11 cm, and the standard deviations are between 1 and 4 cm. The mean distances at horizontal surfaces are in the range in 0–6 cm, and the standard deviation is between 1 and 5 cm. These results are consistent with expectations as they fall close to the manufacturer specification, except they are based on data acquired on a moving platform. Furthermore, it is important to point out that the reference surfaces are not ideal, so they have some contribution to the differences; for example, the vertical surfaces of the buildings could be slightly warped or the roads curvature and unevenness may not be accurately modeled by the quadratic parameters.

## 6. Conclusions

In this study, a test vehicle equipped with several Velodyne sensors as well as a high-performance GNSS/IMU navigation system was used to create a point cloud that can be subsequently used to generate a 3D high-definition map. The georeferencing solution was obtained with the integration of GNSS and IMU data. Using a navigation-grade IMU was an essential contribution to achieving a highly accurate and seamless navigation solution, as the cm-level georeferencing accuracy is critical for the point cloud accuracy evaluation, as the ranging accuracy of the Velodyne sensor is in the few cm range. Seven Velodyne sensors installed in different orientations in the GPSVan collected point clouds in multiple sessions at the OSU main campus. The boresight misalignment parameters of the LiDAR sensors were estimated with calibration test measurements based on LiDAR-specific targets. Using boresighting and georeferencing solution, the point clouds were transformed from the sensor coordinate system to the global coordinate system, and then, the multiple point clouds were fused. In order to assess the accuracy of the point cloud, nine buildings and six road surfaces were selected and measured by conventional surveying methods at cm-level accuracy. These reference point clouds were compared to those obtained from the LiDAR-based point clouds. The mean distances at vertical and horizontal surfaces fall into the ranges of 0–11 cm and 0–4 cm, receptively. In both cases, the standard deviations are between 1–5 cm. This is consistent with the fact that the object range was about 2–3 times larger for the vertical surfaces. The results clearly show that, using a highly accurate georeferencing solution, the point cloud combined from the Velodyne sensors can achieve cm-level absolute accuracy within a 50-m range from a moving platform operating under normal traffic conditions. This performance level is comparable to accuracies that modern mobile mapping systems can achieve, except automotive scanners are used instead of professional grade LiDAR sensors.

## Figures and Tables

**Table 1:** Sensor overview.

Type	Sensor Model	Sensor ID	Location	Sampling Frequency	Angular Resolution H/V	Field of View H/V
GNSS	Septentrio	SEPT	Top	10 Hz	-	-
	PolaRx					
GPS	Novatel DL-4	NOVATEL	Top	5 Hz	-	-
IMU	MicroStrain	MS	Inside	200 Hz	-	-
	3DM-GX3					
IMU	H764G IMU1	H764G	Inside	200 Hz	-	-
IMU	H764G IMU2	H764G	Inside	200 Hz	-	-
LiDAR	Velodyne	VHDL	Front,Top	20 Hz	0.2°/1.33°	360°/40°
	HDL-32E					
LiDAR	Velodyne	VRED	Front,Bottom	20 Hz	2.0°/0.2°	30°/360°
	VLP-16	VBLUE	Back,Center			
LiDAR	Velodyne	VGREEN	Front,Right	20 Hz	0.2°/2.0°	360°/30°
	VLP-16	VYELLOW	Front,Left			
		VWHITE	Back,Left			
		VBLACK	Back,Right			

**Table 2:** Statistical analysis of estimated standard deviations.

		GNSS only				GNSS/IMU			
	East	North (m)	Height	East	North (m)	Height	Roll	Pitch (arcmin)	Heading
min.	0.01	0.01	0.01	0.01	0.01	0.01	0.02	0.02	0.02
average	0.08	0.09	0.20	0.02	0.02	0.02	0.27	0.26	3.48
max.	7.28	4.02	16.97	0.08	0.08	0.09	0.38	0.38	4.13
std.	0.23	0.20	0.61	0.01	0.01	0.02	0.03	0.03	0.25

**Table 3:** Residuals at reference surfaces; vertical (left), and horizontal (right).

Vertical Surface No	Mean Distance	Standard Deviation (m)	Horizontal Surface No	Mean Distance (m)	Standard Deviation (m)
VS-1	0.00	0.02	HS-1	0.01	0.03
VS-2	0.00	0.03	HS-2	0.00	0.01
VS-3	−0.03	0.02	HS-3	−0.06	0.05
VS-4	0.00	0.01	HS-4	0.02	0.02
VS-5	−0.01	0.04	HS-5	0.02	0.03
VS-6	−0.04	0.02	HS-6	−0.02	0.02
VS-7	−0.10	0.01			
VS-8	0.00	0.02			
VS-9	−0.11	0.04			
